# Epidemiologic Features of Acute Pediatric Diarrhea in Managua, Nicaragua, from 2011 to 2019

**DOI:** 10.4269/ajtmh.21-0793

**Published:** 2022-06-15

**Authors:** José Victor Zambrana, Fausto Andres Bustos Carrillo, Sergio Ojeda, Brenda Lopez Mercado, Krista Latta, Amy Schiller, Guillermina Kuan, Aubree Gordon, Arthur Reingold, Eva Harris

**Affiliations:** ^1^ Sustainable Sciences Institute, Managua, Nicaragua;; ^2^ Division of Infectious Diseases and Vaccinology, School of Public Health, University of California, Berkeley, California;; ^3^ Division of Epidemiology and Biostatistics, School of Public Health, University of California, Berkeley, California;; ^4^ Department of Epidemiology, School of Public Health, University of Michigan, Ann Arbor, Michigan;; ^5^ Centro de Salud Sócrates Flores Vivas, Ministry of Health, Managua, Nicaragua

## Abstract

Diarrhea remains a leading cause of death in children in developing countries, including Nicaragua, but little is known about patterns of diarrhea occurrence in Central America over long periods of time. The purpose of this study was to determine the incidence, risk factors, long-term trends, and seasonality of diarrhea in children age 2 to 14 years in Managua, Nicaragua. From 2011 to 2019, we examined episodes of diarrhea among 6,485 children who participated in a prospective cohort study and presented for care in a primary care facility. We performed a longitudinal analysis considering time-varying variables and the intra-subject correlation of outcomes. In addition, we analyzed the weekly incidence of diarrhea, applying seasonal trend decomposition to extract secular and seasonal patterns. The overall incidence rate of diarrhea was 133.4 episodes per 1,000 person-years (95% CI, 128.3–138.7). We observed a slight increase in the incidence of diarrhea from 2011 to 2019. Younger age was the strongest predictor of the risk of diarrhea, and incidence increased with every additional hour without running water in the household per day. Diarrhea incidence in Managua was seasonal, with high peaks each year between May and July. Despite reductions in childhood mortality since 1990 in Nicaragua, diarrheal morbidity remains a major problem in Managua.

## INTRODUCTION

The world has made substantial efforts to reduce mortality in children younger than 5 years of age. In 1990, the global “under-five” mortality rate was 93.2 per 1,000 children, whereas in 2018, the rate had been reduced to 39 per 1,000 children.^1^ Nevertheless, pneumonia, diarrhea, and malaria remain among the leading causes of death in children younger than 5 years old.^1^ In 2016, diarrhea was the fifth leading cause of death in children younger than 5 years old and the second most common cause of years of life lived with disability (YLD) because of its detrimental effects on childhood growth and cognitive development.^2^^,^^3^ Diarrhea is a disease of inequity because it affects the poor and marginalized disproportionately, especially those without access to safe water, hygiene, and health care.^2^^,^^4^ Estimates from the Global Burden of Disease (GBD) Project show that up to 80% of deaths from diarrhea could be feasibly prevented by improvements in access to safe water, sanitation, and better nutrition.^2^

Initiatives such as the Sustainable Development Goals and the Global Action Plan for the Prevention and Control of Pneumonia and Diarrhea have established ambitious plans to reduce childhood mortality to 25 per 1,000 children, and to reduce deaths from diarrhea to less than one per 1,000 children, respectively, by 2030. To track the success of such initiatives, it is essential to monitor changes in diarrheal disease patterns over time, create awareness among policymakers and other stakeholders about the importance of diarrhea to pediatric health, and determine the allocation of resources for effective interventions.^5^

In Nicaragua in 2017, enteric diseases were the leading cause of YLDs in children younger than 5 years old and the fifth most important cause in children 5 to 14 years of age.^6^ However, according to the GBD Project and the World Bank, official Nicaraguan health and vital statistics data have substantial limitations,^7^^,^^8^ making the results of independent epidemiological research especially valuable to Nicaraguan policymaking while helping to monitor and evaluate progress toward global health goals. From 2004 to 2019, the Pediatric Dengue Cohort Study (PDCS) in Managua, Nicaragua, has monitored the health of 9,315 cohort participants. PDCS data are a rich source of longitudinal health information, including the use of medical services and a detailed record of diagnoses. We used this unique data set to examine the incidence of diarrhea in PDCS participants and to characterize changes in a longitudinal framework with regard to the frequency of and factors associated with diarrhea. The objectives of this analysis were to determine the incidence, risk factors, long-term trends, and seasonality of acute diarrhea in PDCS participants in Managua, Nicaragua, from 2011 to 2019. This analysis will inform the Nicaraguan government regarding the health needs among children, compare the observed progress to the findings of the 2019 GBD Project, and contribute to the newly released UN Sustainable Development Goals by providing data to track progress on SDG target 3.9.

## METHODS

### Ethics statement.

The PDCS protocol was reviewed and approved by the institutional review boards of the Nicaraguan Ministry of Health; the University of California-Berkeley; and the University of Michigan. Parents or guardians of all children provided written informed consent, and children ages 6 to 14 years provided verbal assent.

### Study design.

The PDCS is an ongoing, open cohort of ∼3,800 children 2 to 17 years of age based in the Health Center Sócrates Flores Vivas (HCSFV) in District II of Managua, the capital of Nicaragua.^9^^,^^10^ The PDCS captures illnesses among participants through enhanced passive surveillance at the HCSFV. When presenting with fever, participants are screened for arboviral symptoms and signs, and molecular and serological methods are used to confirm arboviral infections. Periodic home visits are made to collect convalescent samples and provide medical follow-up to participants who miss medical appointments. Children who require additional medical care are transferred to the National Pediatric Reference Hospital, Hospital Infantil Manuel de Jesús Rivera. Annually, healthy blood samples are collected, along with weight and height data, and surveys are conducted to update individual and household data.

### Study population and site.

The study health center is part of the Nicaraguan Ministry of Health and serves approximately 61,000 persons in its catchment area. Initial cohort recruitment occurred in 2004 through house-to-house visits, when eligible children were invited to participate. The eligibility criteria were 1) 2 to 9 years of age (in 2007, the upper age limit was extended to 14 years; in 2017, it was extended to 17 years), 2) residence in the HCSFV catchment area with no plans to leave for 3 years, 3) willingness to attend the HCSFV for all primary medical care, 4) no immune-compromising conditions, and 5) parental consent and assent for children older than 5 years. Each year, new 2-year-old children are enrolled to maintain the initial age structure of the cohort. Detailed descriptions of PDCS design and procedures have been published elsewhere.^10^^,^^11^ The current report is restricted to children aged 2 to 14 years enrolled in the PDCS from January 2011 to December 2019.

The PDCS serves neighborhoods along Lake Xolotlán in Managua. About 4% of households have no toilet or latrine and are not connected to a public pipeline network for their water supply.^12^ Approximately 25% and 13% of the population in District II lives in poverty or extreme poverty, respectively, according to the last national census from 2005.^12^

### Survey data.

At enrollment and at every annual sampling, during July from 2004 to 2010 and then during March from 2011 to 2019, a questionnaire was administered to all participants to capture data on demographic and household characteristics, parental information, and chronic health conditions in the children. Weight and height were measured for each participant.^10^ Body mass index (BMI) percentiles were adjusted for gender and age using reference data from the World Health Organization (WHO).^13^^,^^14^ We evaluated age, sex, time without water, and BMI as risk factors for the incidence of diarrhea. This analysis was restricted to those children with completed household surveys during the same year as the clinical visits (97% of all diarrhea cases). An additional subset analysis was implemented in those with complete BMI measurements who were not severely thin or underweight (BMI-for-age-and-sex Z-score < 2 SDs), as only 1.8% of all BMI measurements were consistent with severe undernourishment.

### Case definition.

Diarrhea cases in the study population were captured through enhanced passive surveillance. Upon presentation to the health center, participants are examined medically by the study physician, who records medical visit data on digital forms that contain more than 150 variables for vital signs, symptoms, treatments, and so on.^15^ These data were used to classify diagnoses and inform the analyses. The diagnostic categories for PDCS medical data, including the primary medical conditions of interest, were coded based on Nicaraguan health regulations and the WHO Protocol for Integrated Management of Childhood Illness.^16^ To permit comparisons with the results of other studies, we recoded all acute diarrhea episodes to correspond to the International Classification of Diseases, 10th revision (ICD-10), version 2016.^17^ Given the limited capacity to confirm underlying diarrheal etiologies by laboratory-based measures at the study health center (as in other primary health-care facilities in Nicaragua), most intestinal infections of PDCS participants were diagnosed clinically. Hence, this analysis was based on clinical diagnoses of diarrhea.

Acute diarrhea episodes were defined by clinical consults, with the primary reason for the visit as one of the following categories: diarrhea without dehydration, diarrhea with dehydration, diarrhea with severe dehydration, and dysenteric diarrhea (gastroenteritis resulting in bloody diarrhea witnessed by a clinician). Episodes of gastroenteritis diagnosed as chronic or persistent diarrhea (diarrhea lasting more than 7 days), vomiting, or unspecified intestinal parasitic infections (ICD-10 B82) listed as the primary reason for the visit were excluded (*n* = 1,921). All the included diarrhea episodes (*n* = 4,424) fall into the ICD-10 category A00–A09, “Intestinal infectious diseases,” specifically in the “Other and unspecified gastroenteritis and colitis of infectious origin” section (ICD-10 A09) and “Gastroenteritis and colitis of unspecified origin” (ICD-10 A09.9) subcategory.

### Follow-up time.

We considered both the first diarrhea episode and recurrent diarrhea episodes. The person-time at risk for the first episode of acute diarrhea was calculated from either January 1, 2011 or the date of enrollment to the first diarrhea episode. A washout period of 20 days between diarrhea episodes was instituted to exclude gastrointestinal episodes and person-time immediately following the initial episode. The person-time at risk for subsequent episodes was calculated from 20 days after the previous episode until a following episode, participant withdrawal from the study, study termination, date of the participant’s 15th birthday, or participant death. When the date for study withdrawal was uncertain, the estimated exit date was calculated as the median date between either the closest contact date or the date of the last-conducted questionnaire (whichever was more recent) and the date when the participant no longer met the inclusion criteria from the PDCS.

### Incidence rate and risk factor analysis.

Intercept-only negative binomial generalized linear mixed models (GLMM) were used to estimate crude rates of diarrhea and 95% confidence intervals (CI). Direct standardization of the overall incidence rate by age and week was performed using Nicaragua’s last census data from 2005. GLMMs were used to estimate incidence rate ratios while also accounting for changes in covariate values over time, intra-participant variability, and recurrent events. GLMMs used the participants’ identifiers as the random effect (intercept) term, were estimated via maximum likelihood, and used Wald’s method to calculate the 95% CI. A literature search guided the decision concerning which variables to include in the multivariable model. A sensitivity analysis was performed to evaluate whether other modeling approaches for estimating measures of incidence differed from the main risk factor analysis (Supplemental Figure S3). The 31 participants with more than five episodes of acute diarrhea exerted substantial influence in the final model; as a result, they were removed from the analysis.

To characterize the functional relationship between the incidence of diarrhea and age, and between incidence of diarrhea and daily hours without water in the household, negative binomial mixed-effects generalized additive models were used,^18^^,^^19^ with thin-plate regression splines and restricted maximum likelihood used to select the smoothing parameter. Generalized additive models were adjusted for gender, used participant identifiers as the random effect (intercept) term, and included log follow-up time as the offset term. Robust 95% CIs were calculated semiparametrically for plotting smooth effects.^18^

### Time trend and seasonality.

Crude incidence rates were calculated by weekly intervals based on the actuarial life-table method to explore patterns and trends in the time series of the PDCS population. Seasonal trend decomposition by locally estimated scatterplot smoothing (LOESS)^20^ was used to extract smooth estimates of trend (*T*), seasonality (*S*), and the residuals (*R*, the model errors), from the observed time series line (*Y*) over time (*t*):

$$Y_{t}\;=\;T_{t}+S_{t}+R_{t}.$$

The time trend model was parameterized with a non-periodic window for the seasonal component to account for changes in seasonality, and the trend component used a moving average of 2 years to evaluate long-term trends.

All analyses were conducted in R version 5.6.3 (R Foundation for Statistical Computing, Vienna, Austria). The major R packages for this project were *lme4* (v 1.1-27.1), *survival* (v3.2-11), *mgcv* (v1.8-36), *tidyverse* (v1.3.1),^21^^–^^23^ and *stats* (v4.2.0).^24^ The R scripts generated for the analysis can be accessed at https://github.com/josviczammad2/Epidemiologic-features-of-acute-pediatric-diarrhea-in-Managua.

## RESULTS

### Participant characteristics.

A total of 6,485 children contributed 28,329 person-years to the study. Yearly participation in the PDCS ranged from 2,945 to 3,531 children (Table 1). The median participation time per child was 5.11 years (interquartile range, 1.99–6.15 years). On average, 434 new participants were enrolled every year to maintain the cohort’s age structure (Supplemental Figure S1). The distribution of gender and age groups was very similar across all years. Throughout the study period, participants’ households had an increase in daily hours with water supply (Table 1). The best year of household water supply was 2018, when 78% of households had uninterrupted service, 12% of households experienced 1 to 7 hours of interrupted service, and only 10% of households experienced > 7 hours without running water.

**Table 1:** Characteristics of study participants

Category	Calendar year								
	2011	2012	2013	2014	2015	2016	2017	2018	2019
No. of participants	3,015	3,034	2,945	3,352	3,346	3,457	3,428	3,399	3,531
Age, years; *n *(%)									
2–5	879 (0.29)	1,010 (0.33)	936 (0.32)	1,199 (0.36)	1,246 (0.37)	1,299 (0.38)	1,261 (0.37)	1,251 (0.37)	1,279 (0.36)
6–9	1,034 (0.29)	992 (0.33)	946 (0.32)	1,067 (0.36)	1,092 (0.37)	1,164 (0.38)	1,165 (0.37)	1,136 (0.37)	1,175 (0.36)
10–14	1,102 (0.37)	1,032 (0.34)	1,063 (0.36)	1,086 (0.32)	1,008 (0.30)	994 (0.29)	1,002 (0.29)	1,012 (0.30)	1,077 (0.31)
Gender, *n *(%)									
Female	1,517 (0.50)	1,529 (0.50)	1,482 (0.50)	1,688 (0.50)	1,672 (0.50)	1,727 (0.50)	1,704 (0.50)	1,694 (0.50)	1,760 (0.50)
Male	1,498 (0.50)	1,505 (0.50)	1,463 (0.50)	1,664 (0.50)	1,674 (0.50)	1,730 (0.50)	1,724 (0.50)	1,705 (0.50)	1,771 (0.50)
No. of hours without water, daily; *n *(%)									
0	1,537 (0.51)	1,486 (0.49)	2,252 (0.76)	2,344 (0.70)	2,439 (0.73)	2,569 (0.74)	2,285 (0.67)	2,655 (0.78)	2,632 (0.75)
1–7	512 (0.17)	627 (0.21)	320 (0.11)	420 (0.13)	453 (0.14)	419 (0.12)	514 (0.15)	407 (0.12)	435 (0.12)
> 7	966 (0.32)	921 (0.30)	373 (0.13)	588 (0.18)	454 (0.14)	469 (0.14)	629 (0.18)	337 (0.10)	464 (0.13)
Body mass index,* *n *(%)									
Normal weight	2,295 (0.80)	2,218 (0.79)	1,661 (0.77)	1,254 (0.80)	1,587 (0.79)	2,482 (0.76)	2,456 (0.74)	2,331 (0.74)	2,471 (0.73)
Overweight	356 (0.12)	390 (0.14)	313 (0.14)	191 (0.12)	229 (0.11)	474 (0.14)	516 (0.16)	488 (0.15)	514 (0.15)
Obese	202 (0.07)	212 (0.08)	197 (0.09)	128 (0.08)	184 (0.09)	329 (0.10)	355 (0.11)	345 (0.11)	401 (0.12)
Incidence of diarrhea cases over time									
No. of cases	369	444	338	558	606	600	574	462	473
Person-years	2,852.4	2,978.4	2,947.5	3,088.7	3,145.7	3,301.8	3,301.4	3,322	3,353.2
Crude IR, 1,000 py	129.4	149.1	114.7	180.7	192.6	181.7	173.9	139.1	141.1
Adjusted IR, 1,000 py	135.3	148.1	110.3	174.1	183.6	168.4	160.5	131.2	132.9

IR = incidence rate; py = person-years.

* Subset analysis.

### Diarrhea incidence.

A total of 4,424 cases of acute diarrhea yielded a modeled incidence rate of 133.4 events per 1,000 person-years (95% CI, 128.3–138.7). The incidence of diarrhea decreased with increasing age (Table 2). Age-standardizing the crude incidence rates using Managua’s last census data resulted in very similar estimates (Table 1, Figure 1, Supplemental Figure S2). We recorded 1,435 participants (21.9%) experiencing one diarrhea episode, 634 (9.7%) experiencing two diarrhea episodes, and 487 (7.4%) experiencing three to five diarrhea episodes across their follow-up time (median, 1 episode; interquartile range, 1–2 episodes). Although the crude incidence rate varied from year to year, ranging from 114.7 to 192.6 per 1,000 person-years, the trend was relatively flat across the 9 years of study, with a sharp increase in 2013 through 2015 that was followed by a return to its average value shortly thereafter (Table 1, Figure 1B).

**Table 2:** Incidence of and risk factors for diarrhea incidence in children in Managua, Nicaragua, 2011 to 2019

Category	No. of cases	No. of py	Crude analysis, GLMM* (crude IR, 1,000 py)	Univariable analysis, GLMM		Multivariable analysis, GLMM		Multivariable analysis, GLMM; with BMI†	
				IRR	*P* value	IRR	*P* value	IRR	*P* value
Overall	4,424	28,329	133.4 (128.3–138.7)	–	–	–	–	–	–
Age, years									
2–5	2,481	8,550	247.4 (234.9–260.5)	**2.3 (2.2–2.5)**	**< 0.001**	**2.3 (2.2–2.5)**	**< 0.001**	**2.4 (2.2–2.6)**	**< 0.001**
6–9	1,211	9,784	103.8 (98.2–109.8)	Ref.	–	Ref.	–	Ref.	–
10–14	732	9,996	62.4 (58.4–66.6)	**0.6 (0.5–0.6)**	**< 0.001**	**0.6 (0.5–0.6)**	**< 0.001**	**0.6 (0.5–0.6)**	**< 0.001**
Gender									
Female	2,192	14,215	131.6 (124.8–138.7)	Ref.	–	Ref.	–	Ref.	–
Male	2,232	14,114	135.2 (127.8–143.1)	1.0 (1.0–1.1)	0.494	1.0 (1.0–1.1)	0.52	1.0 (0.9–1.1)	0.571
No. of hours without water, daily									
0	3,023	19,765	129.2 (123.6–135.0)	Ref.	–	Ref.	–	Ref.	–
1–7	607	3,861	139.1 (128.3–150.8)	**1.1 (1.0–1.2)**	**0.018**	1.1 (1.0–1.2)	0.178	1.1 (1.0–1.2)	0.107
8–24	794	4,703	148.2 (137.6–159.5)	**1.2 (1.1–1.3)**	**< 0.001**	**1.2 (1.1–1.3)**	**< 0.001**	**1.2 (1.1–1.3)**	**< 0.001**
Body mass index									
Normal weight	3,291	19,006	147.2 (140.8–153.9)	Ref.	–	–	–	Ref.	–
Overweight	453	3,658	108.8 (97.5–121.4)	**0.7 (0.6–0.8)**	**< 0.001**	–	–	1.0 (0.9–1.1)	0.996
Obese	344	2,512	116.3 (103.5–130.7)	**0.7 (0.6–0.8)**	**< 0.001**	–	–	1.1 (1.0–1.2)	0.156

BMI = body mass index; GLMM = generalized linear mixed model; IR = incidence rate; IRR = incidence rate ratio; py = person-years; Ref. = reference category. Covariates in bold type are statistically significant.

* GLMM rates account for inter-participant variability.

† Subset analysis.

**Figure:**
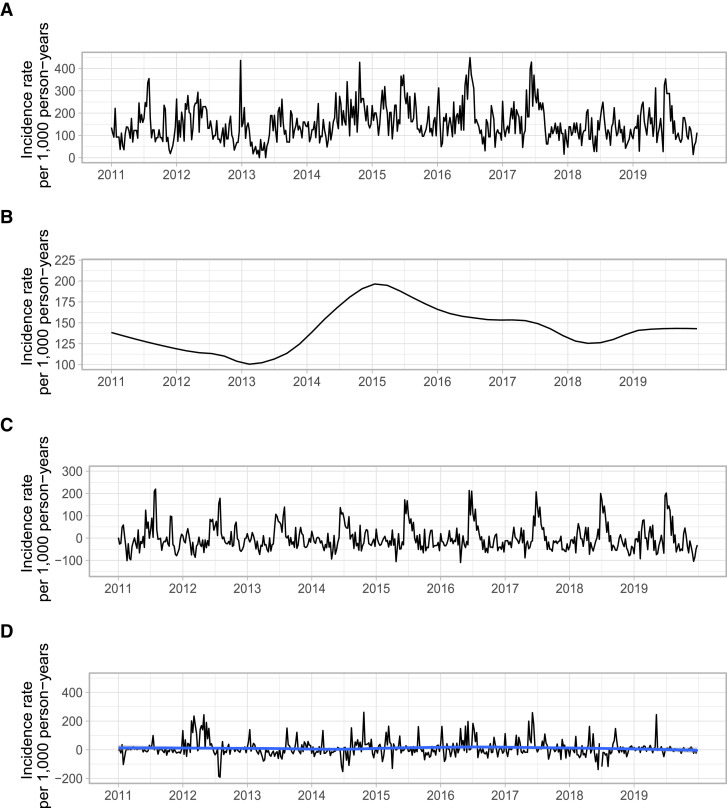
Figure 1. Seasonal trend decomposition of the acute diarrhea incidence rate by epidemiological week. Time–trend decomposition by LOESS of the incidence rate of acute diarrhea per 1,000 person-years. (**A**) Raw incidence rate. (**B**) Underlying trend. (**C**) Seasonal component. (**D**) Residual (Model error = Raw data – Underlying trend – Seasonal component). The blue line is an estimate of the residual trend by locally estimated scatterplot smoothing (LOESS). This figure appears in color at www.ajtmh.org.

### Risk factor analysis.

Individual- and household-level risk factors for acute diarrhea were analyzed. Younger age was strongly associated with diarrhea across the study period; the incidence of diarrhea decreased significantly with increasing age (Table 2). With increasing age, the model-predicted incidence rate of diarrhea occurrence decreased (Figure 2). The steepest slope was between 2 to 4 years of age, followed by a period of slow decline and a subsequent sharp decrease between 10 to 14 years of age. Male children had a slightly greater crude incidence rate of diarrhea compared with female children, but in both univariable and multivariable analyses, there was no evidence of a difference in the incidence rate by gender. Higher BMI categories were associated with a decreased incidence of diarrhea in the univariable, but not the multivariable, analysis.

**Figure:**
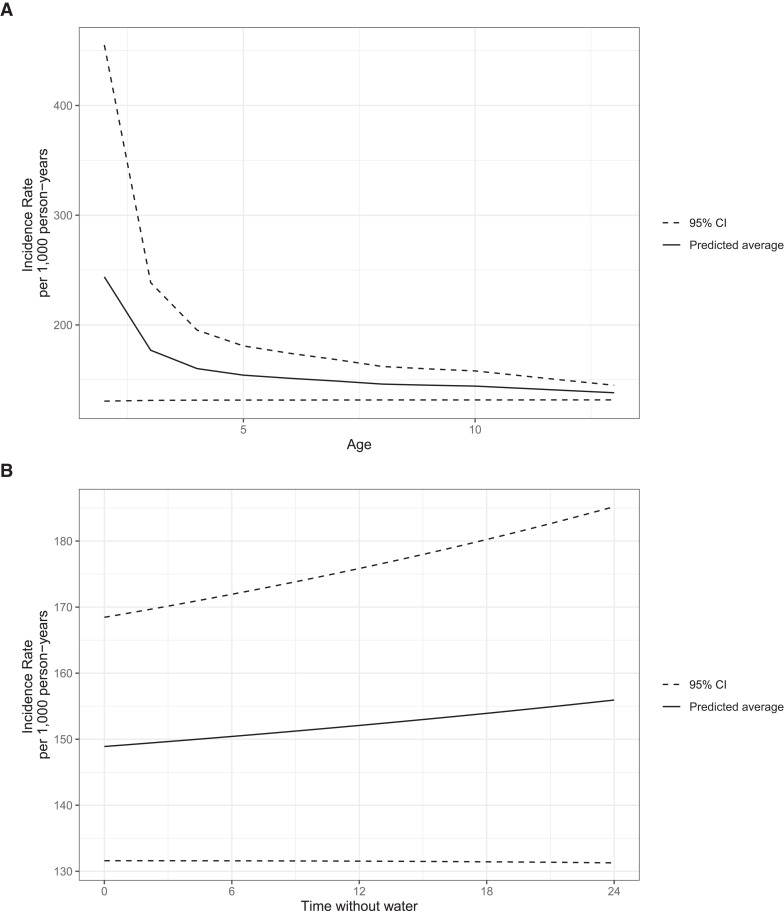
Figure 2. Incidence rates of acute diarrhea per 1,000 person-years by age and hours without water. (**A**) Effects of age. (**B**) Effects of hours without water. Trend lines were estimated using generalized additive modes, which do not assume the modeled relationships have a linear form.

In a multivariable analysis, participants who reported at least 8 hours per day without water supply in the home had an incidence rate of diarrhea 1.17 times greater than participants in homes having an uninterrupted water supply during the day (Table 2). A linear relationship between the hours without water in the home and the incidence rate of diarrhea at the individual level was found, holding other covariates fixed (Figure 2).

We performed the same risk factor analysis with other statistical models appropriate for count data, all of which exhibited similar goodness-of-fit metrics. Predictions from alternative models were very similar to the main results (Supplemental Figures S3 and S4).

### Seasonal and trend analysis.

Episodes of acute diarrhea exhibited marked seasonality, with cases peaking between May and July annually, except in 2013 and 2014, and with secondary peaks each January since 2017 (Figure 1C). The incidence of diarrhea was lowest in December in most years. The crude incidence rate of diarrhea increased from 100 cases per 1,000 person-years in 2013 to a weekly average of 200 cases per 1,000 person-years in 2015, and then slowly decreased (Figure 1B). In 2019, the last year of observation, the underlying trend of diarrhea incidence plateaued at 137 cases per 1,000 person-years. Crude incidence rates stratified by the different covariates showed similar trends over time, but the seasonal component was less clear for children with a greater risk of diarrhea—namely, children aged 2 to 5 years and children living with an interrupted water supply (Supplemental Figures S5–S8).

## DISCUSSION

We analyzed the incidence, risk factors, temporal trends, and seasonal patterns of acute diarrhea among 6,548 children in a longitudinal cohort study in Nicaragua over 9 years. We found a high frequency of diarrhea among all age groups, averaging 133.4 episodes per 1,000 person-years in our crude model. The highest incidence rates were experienced by children 2 to 5 years of age, with decreasing incidence among older children.

Our results are consistent with those of multiple prior studies showing a reduction in the incidence of diarrhea with increasing age.^1^^,^^2^^,^^25^^,^^26^ In 2012, one research group estimated that, on average, Latin American children younger than 5 years old experienced four episodes of diarrhea per year from 1990 to 2010 (4,000 cases per 1,000 person-years),^27^ which is ∼14 times greater than the overall rate we observed among 2- to 5-year-old PDCS participants (247.4 cases per 1,000 person-years in crude model incidence). This difference could be explained by our urban study population, by the age distribution in our cohort, or because we included only episodes of medically attended acute diarrhea. However, the authors of that review mentioned that their 2010 estimates were predominantly based on older data because of a lack of more recent cohort studies.^27^ Studies such as ours provide current estimates of the incidence of diarrhea in Central America. Becker-Dreps et al.^28^ found similar estimates to ours for the incidence of diarrhea in Léon, Nicaragua’s second largest city. During a 3-year period (2007–2009), they found an incidence rate of diarrhea between 74 and 164 per 1,000 children 1 to 5 years of age.^28^ Our study and that of Becker-Dreps et al.^28^ found high incidence rates of pediatric diarrhea, and both studies found a lower diarrhea incidence of diarrhea in Nicaragua than that experienced across Latin America in general.^27^

We did not find a significant association between the rate of diarrhea episodes and gender, although we observed a slightly greater incidence rate among male children. The associations between diarrhea and gender in prior studies have varied, but most studies have found a greater incidence or prevalence of diarrhea in male children compared with female children.^25^^,^^29^ For instance, a study of 14,500 children older than 5 years of age across five Mesoamerican countries, including Nicaragua, found a significant association between the prevalence of diarrhea and gender, with male children having 1.15 greater prevalence odds of diarrheal disease compared with female children, after adjusting for socioeconomic status, age, water treatment, and sanitation.^29^

It is well known that diarrhea can cause and is an effect of malnutrition among children.^30^ There were far too few underweight children in our study population to estimate diarrhea incidence robustly in that BMI category. However, we tested whether overweight and obese children had a greater incidence of diarrhea compared with normal-weight children, and found that overweight and obese children do not have a greater incidence of acute diarrhea cases. Although obesity is a risk factor for chronic gastrointestinal diseases, a granular analysis of the impact of BMI and metabolic measures on the incidence of diarrhea was beyond the scope of our study.^31^

We found a positive association between the number of daily hours without water in the home and the incidence of diarrhea. Similar studies have found the same association but interrupted piped water access is defined differently across studies,^32^^–^^35^ making direct comparisons difficult. To our knowledge, our study is the first to document the association between intra-day water supply access on an hourly basis and the incidence of acute diarrhea. Explanatory mechanisms underlying the relationship between intermittent water access and indicators of microbial quality of piped water have been described previously; they include water stagnation, pipe drainage, intrusion of sediment into the pipes, backflow, first flush events (i.e., restarting water supply), and storage of water at the household level.^36^^–^^38^ In our study, improvements in the reliability of household water supply were observed during the study period. However, our results suggest that a consistent water source is necessary to have the full benefits of an improved household water supply.

The incidence of diarrhea demonstrated a seasonal pattern throughout the study period, showing a large peak between May and July, with ∼200 additional cases of diarrhea per 1,000 person-years from 2015 through 2017 compared with the baseline seasonal effect. Although we lack data on the etiology of diarrhea episodes, studies in Léon, Nicaragua, found the most frequent etiologic agents responsible for diarrhea were norovirus and sapovirus, accounting for up to 45% of all diarrhea episodes.^39^^,^^40^ A similar seasonal pattern to the one we identified was observed in Léon, where high peaks of norovirus-induced gastroenteritis occurred between May and the end of July, although the virus itself circulated throughout the year.^39^^,^^41^ Yearly rainfall in Nicaragua has a bimodal distribution; it is highest in May and next highest between September and October.^42^^,^^43^ Time-series analyses from around the globe have found an association between the incidence of diarrhea and precipitation, including norovirus circulation,^44^^–^^48^ which could explain the high peaks of cases of diarrhea in the middle of the year, but not the low incidence observed during September and October.

The GBD Project estimates from 2011 to 2017 for Nicaragua show an increase of 7.89% in the YLDs resulting from diarrheal diseases for children between 5 and 14 years of age, and almost no change for children younger than 5 years of age. Over the same time period, GBD Project estimates for Nicaragua also showed reductions in mortality of –23.24% and –37.62% as a result of diarrheal disease in children 5 to 14 years and less than 5 years of age, respectively.^6^^,^^49^ Thus, although diarrhea-associated pediatric mortality has recently decreased in Nicaragua, morbidity has increased despite improvements in disease management with oral rehydration therapy,^50^ the introduction of the rotavirus vaccine,^51^ and greater coverage of essential health services.^52^ Taken together, the GBD Project estimates and our results suggest that diarrhea-associated morbidity has not declined appreciably since 2017. The documented reductions in mortality might be a result of changes in disease management or health-care access, not a result of a reduction in diarrhea-related exposures, which usually requires large investments in water purification and sanitation infrastructure.^52^

Our study has many strengths, including its high-quality data collection and quality control procedures, large sample size, high participation rate, long duration of follow-up, and low proportion of censored participants. Our study also had several limitations. First, no etiological data were available for this study. Second, unobserved covariates such as socioeconomic status and hygiene could explain more of the variability in the outcome than we were able to measure. Third, the PDCS does not enroll newborns and 1-year-old children, which is when most diarrhea cases occur. Last, health-care use and incidence of diarrhea might be different in smaller cities and rural Nicaragua, so our results may not generalize to the entire country.

This study documented the high burden of diarrheal disease in children in Managua, showing that age, the number of daily hours without access to water in the household, and season were strongly associated with the incidence of diarrhea. High-quality longitudinal data on medically attended illnesses are rarely available from Central American nations. The results of this study will benefit the Nicaraguan Ministry of Health, as well as the Nicaraguan National Aqueduct and Sewer Company, when implementing future policies aimed at improving the health of Nicaraguan children. More broadly, this study strengthens calls to implement uninterrupted water supplies as a regional and global policy to reduce the substantial morbidity and mortality associated with pediatric diarrhea.

## Supplemental Material


Supplemental materials
