# Supplementary material for: Epidemiologic Features of Acute Pediatric Diarrhea in Managua, Nicaragua, from 2011 to 2019
Source: Am J Trop Med Hyg. 2022 Jun 15;106(6):1757–64. doi: 10.4269/ajtmh.21-0793 (PMC9209918; doi:10.4269/ajtmh.21-0793)
Supplement: Supplementary file 1 [file tpmd210793.SD1.pdf]

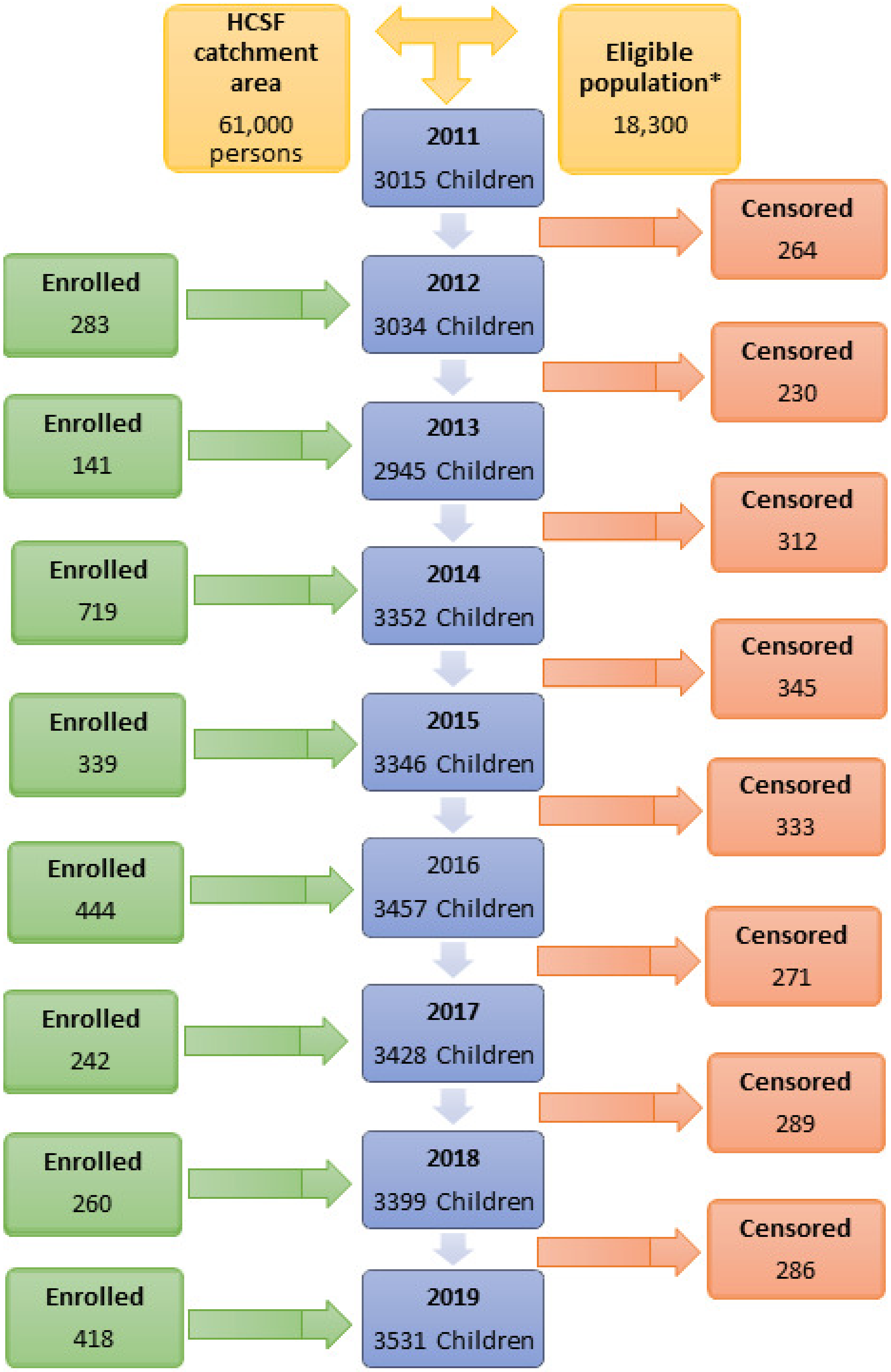

***Proxy based on census data.**[1]

Supplemental Figure 1. Pediatric Dengue Cohort Study participant flowchart. The term *censored* is used as a catch-all term to indicate administrative censoring (participant no longer meets the criteria to be in the study), loss to follow-up, and participant withdrawal from the study.

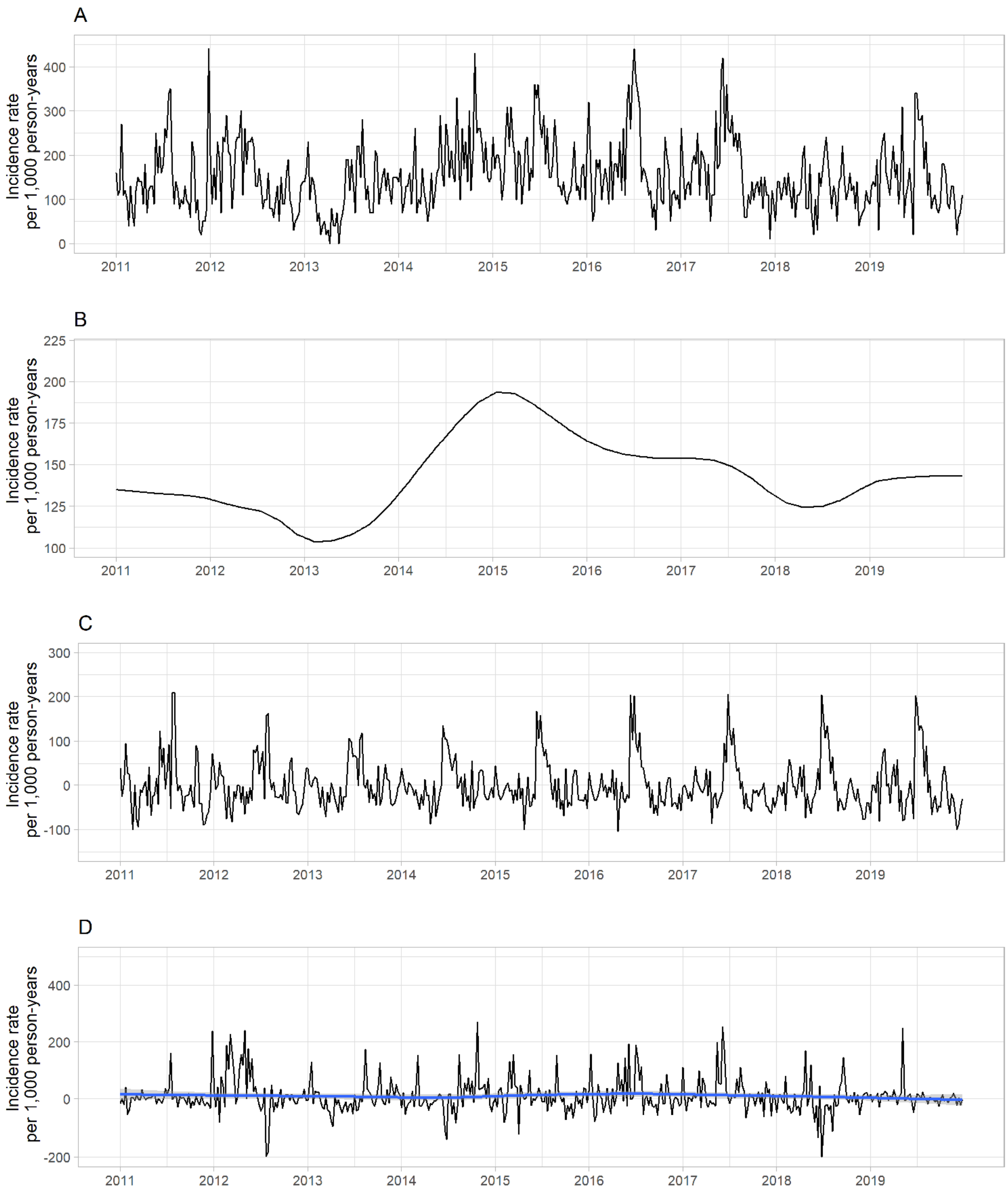

Supplemental Figure 2. Seasonal trend decomposition of age-standardized incidence rates by epidemiological week. Time-trend decomposition by LOESS of the age-standardized incidence rates of acute diarrhea per 1,000 person-years was performed using Managua's age population structure. Panels show **(A)** the raw incidence rate, **(B)** the underlying trend, **(C)** the seasonal component, and **(D)** the residual (model error = raw data – underlying trend – seasonal component). The blue line represents the shape of the residuals by LOESS.

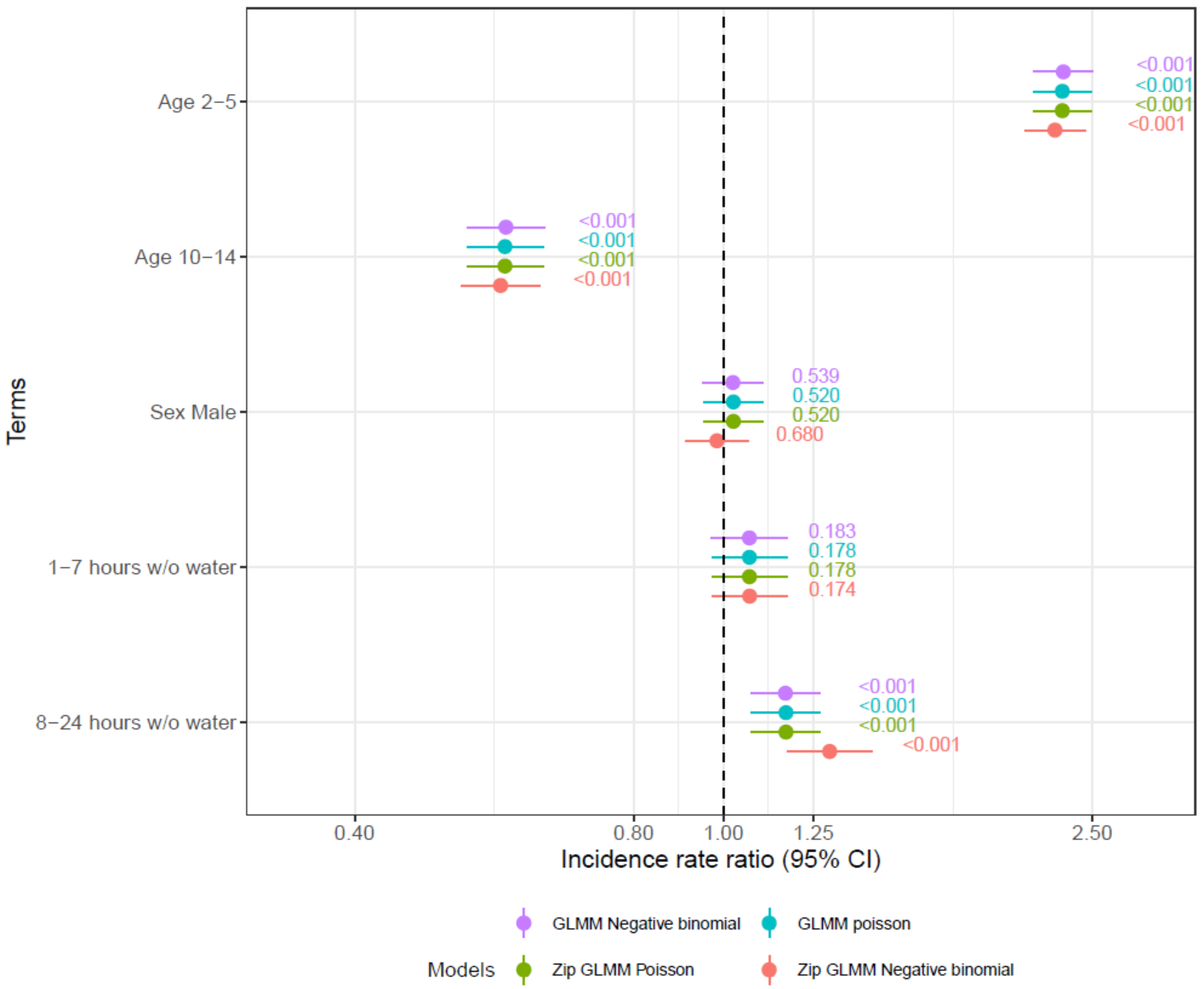

Supplemental Figure 3. Sensitivity analysis of the main risk factor model. The main model is the GLMM negative binomial shown in purple. Alternative models are shown in aqua, light green, and orange, as indicated. P-values for the corresponding covariates are expressed to the right of every estimate.

Abbreviations: GLMM: Generalized linear mixed model; Zip: Zero-inflated

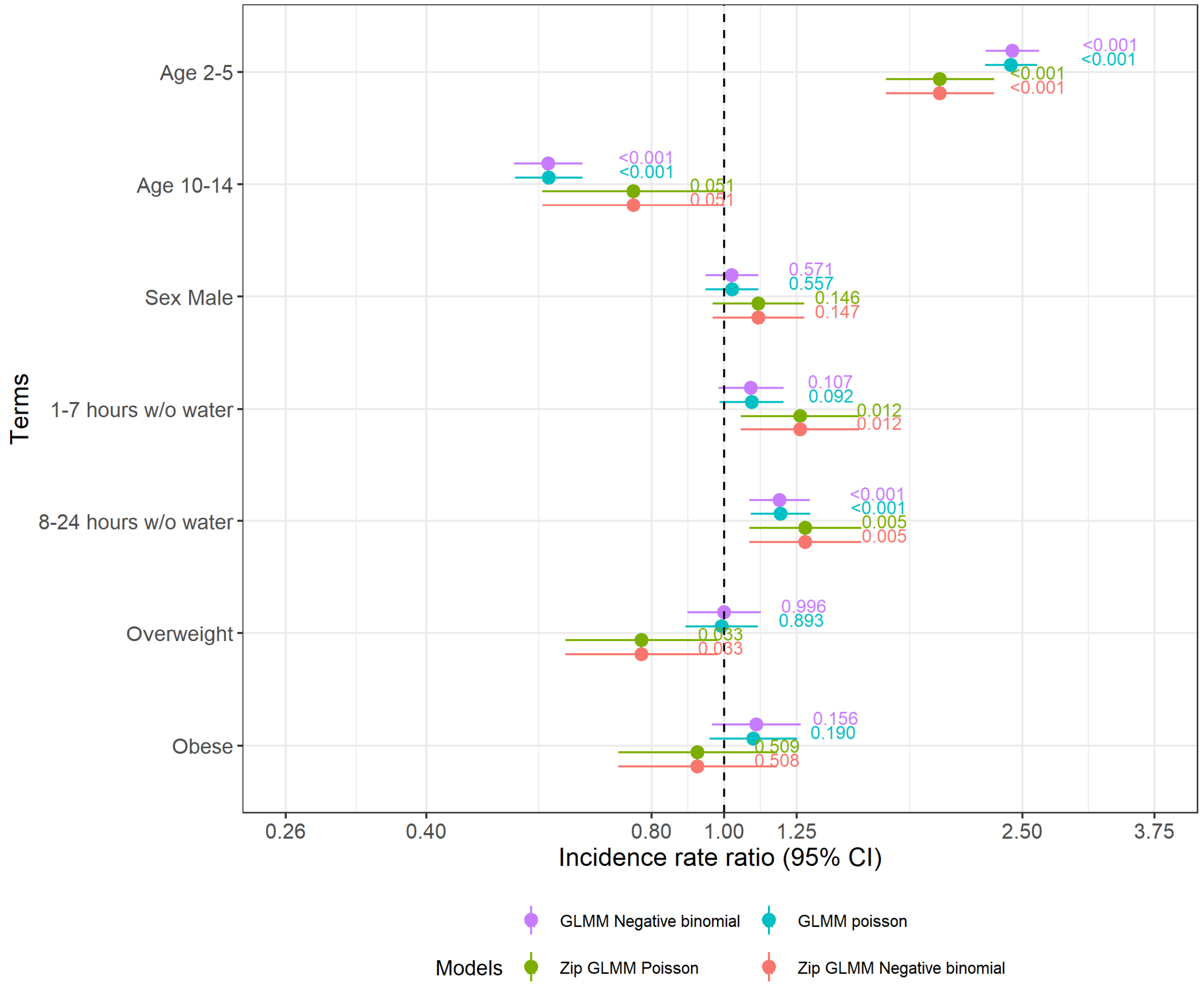

Supplemental Figure 4. Sensitivity analysis of the main risk factor model – subset analysis. The main model is the GLMM negative binomial shown in purple. Alternative models are shown in aqua, light green, and orange, as indicated. P-values for the corresponding covariates are expressed to the right of every estimate.

Abbreviations: GLMM: Generalized linear mixed model; Zip: Zero-inflated

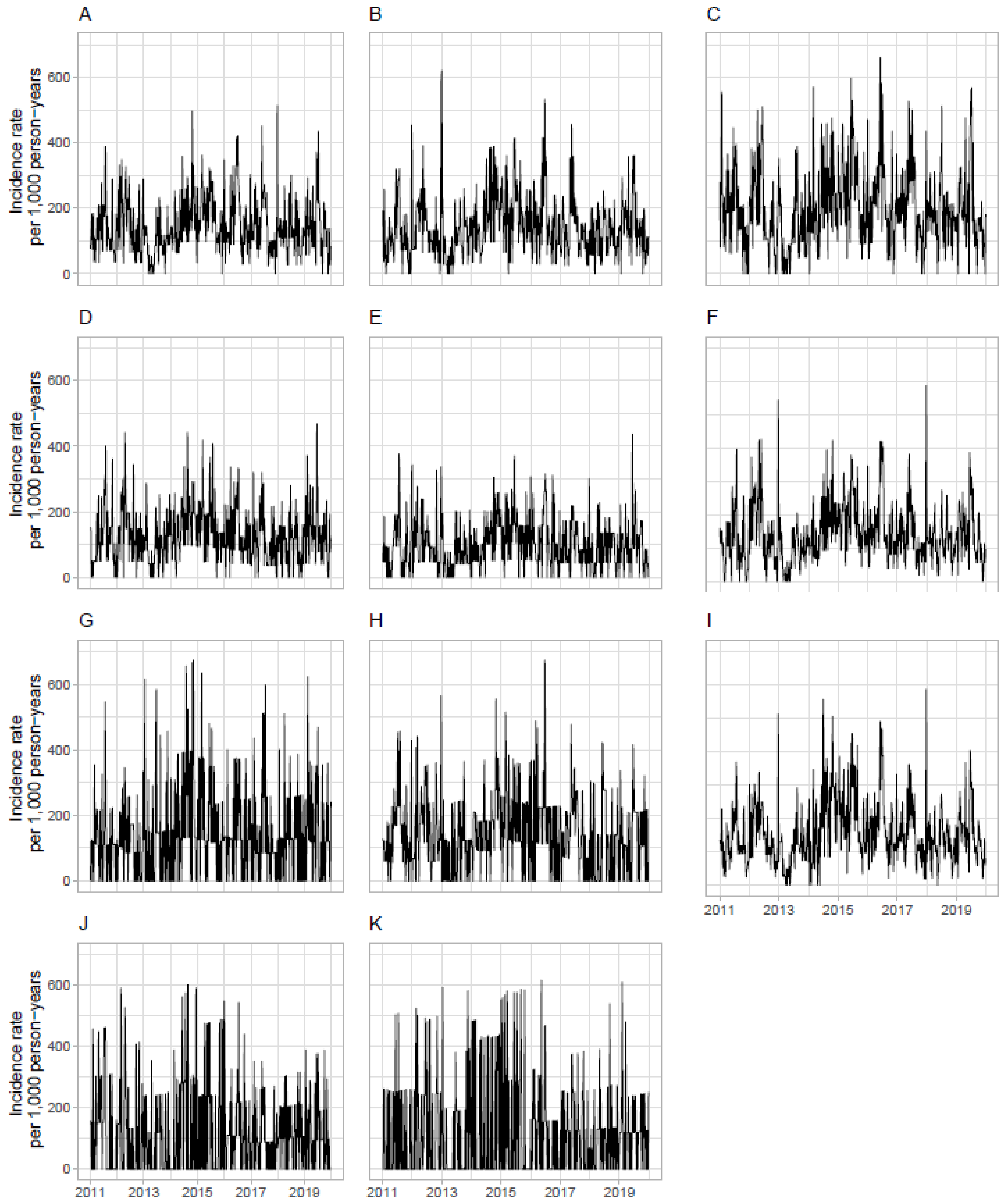

Supplemental Figure 5. **Crude incidence rates by epidemiological week and covariates.** Crude incidence rates by epidemiological week and covariates are shown. Panels show stratification by **(A)** female sex, **(B)** male sex, **(C)** 2-5 years old, **(D)** 6-9 years old, **(E)** 10-14 years old, **(F)** 0 daily hours without water, **(G)** 1-7 daily hours without water, and **(H)** >7 daily hours without water.

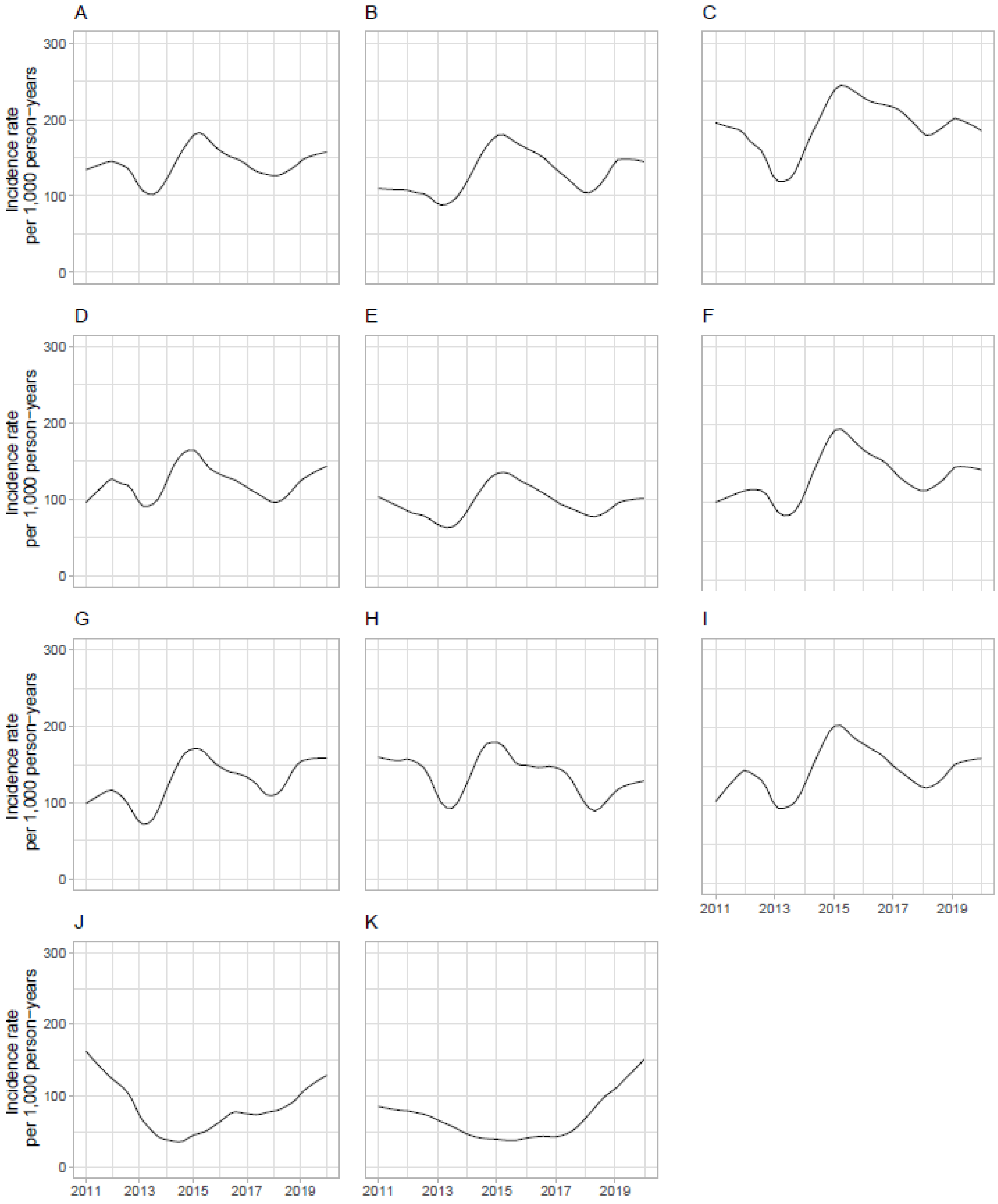

Supplemental Figure 6. **Underlying component of the seasonal trend decomposition of crude incidence rates by epidemiological week and covariates.** Panels show stratification by **(A)** female sex, **(B)** male sex, **(C)** 2-5 years old, **(D)** 6-9 years old, **(E)** 10-14 years old, **(F)** 0 daily hours without water, **(G)** 1-7 daily hours without water, and **(H)** >7 daily hours without water.

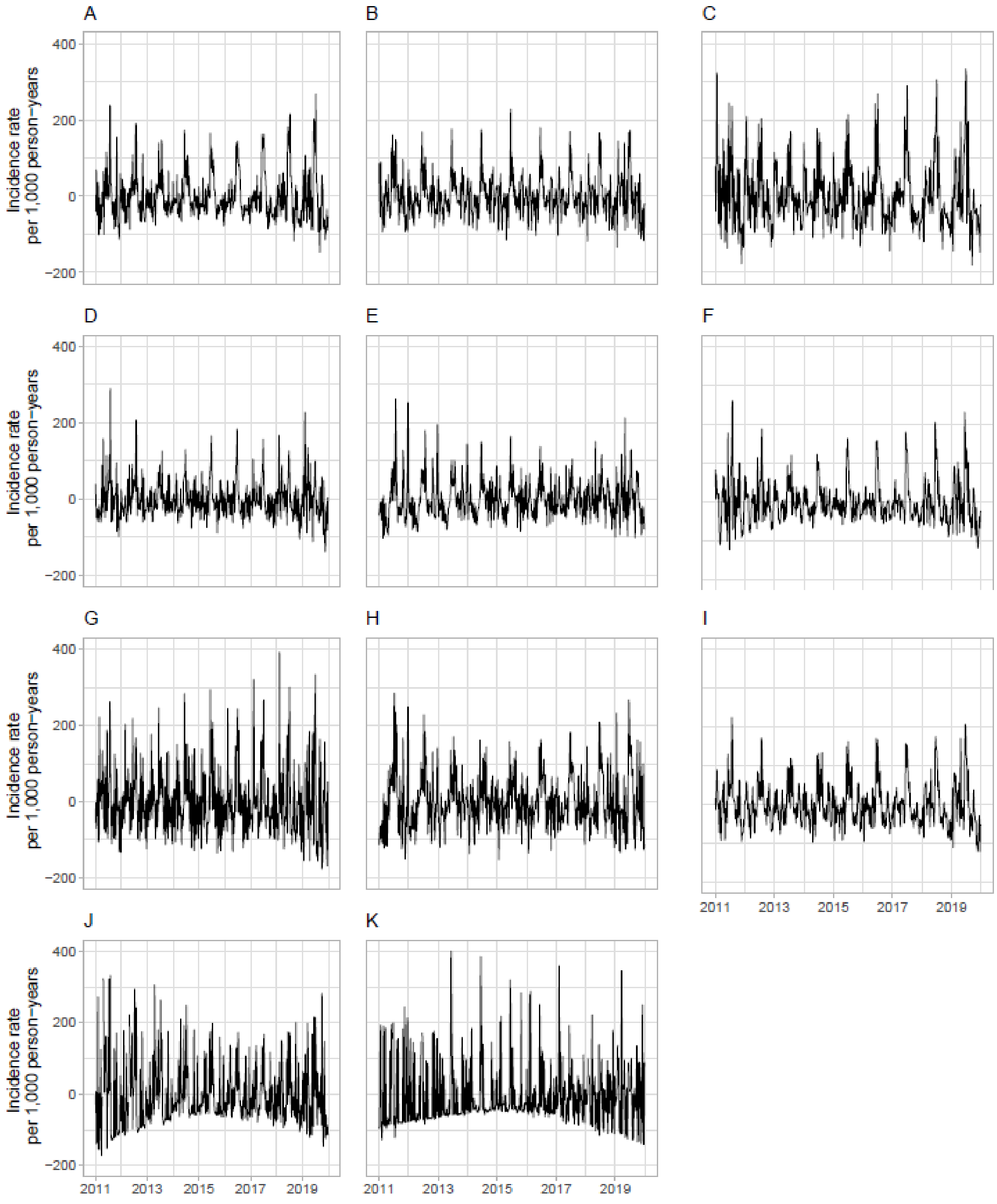

Supplemental Figure 7. Seasonal component of the seasonal trend decomposition of crude incidence rates by epidemiological week and covariates. Panels show stratification by **(A)** female sex, **(B)** male sex, **(C)** 2-5 years old, **(D)** 6-9 years old, **(E)** 10-14 years old, **(F)** 0 daily hours without water, **(G)** 1-7 daily hours without water, and **(H)** >7 daily hours without water.

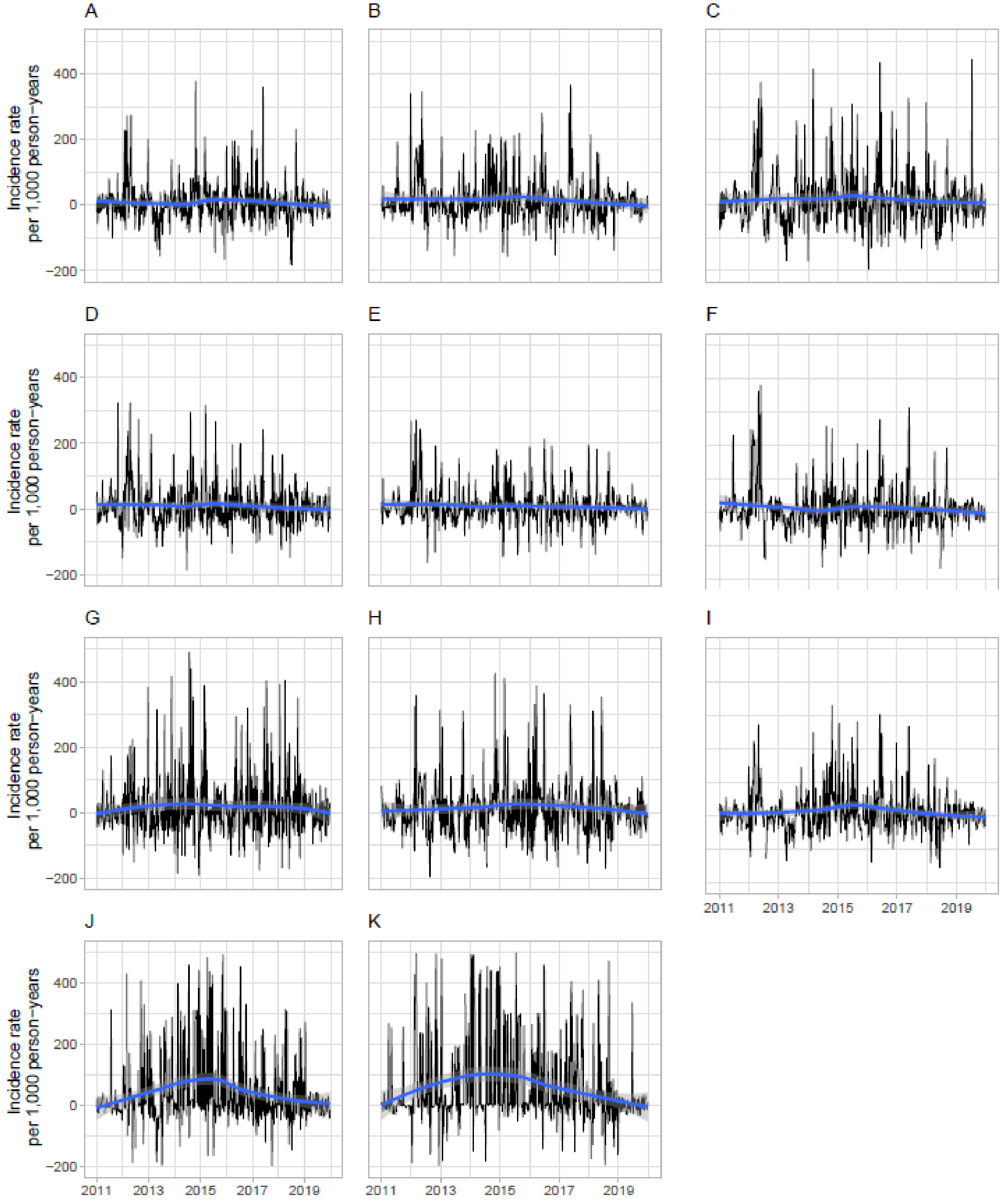

Supplemental Figure 8. Residual component of the seasonal trend decomposition of crude incidence rates by epidemiological week and covariates. Panels show stratification by **(A)** female sex, **(B)** male sex, **(C)** 2-5 years old, **(D)** 6-9 years old, **(E)** 10-14 years old, **(F)** 0 daily hours without water, **(G)** 1-7 daily hours without water, and **(H)** >7 daily hours without water. The blue line represents the shape of the residuals by LOESS.

Reference

1. National Institute of Statistics and Censuses of Nicaragua., 2006. VIII population census and IV household census. Available at: https://www.inide.gob.ni/docu/censos2005/VolPoblacion/Volumen%20Poblacion%201-4/Vol.I%20Poblacion-Caracteristicas%20Generales.pdf. Accessed June 15, 2021.
